# Research progress on plant noncoding RNAs in response to low-temperature stress

**DOI:** 10.1080/15592324.2021.2004035

**Published:** 2021-12-19

**Authors:** Chenmin Huo, Baowen Zhang, Ruiju Wang

**Affiliations:** a College of Biology Science & Engineering, Hebei University of Economics & Business, Shijiazhuang, China; b Ministry of Education Key Laboratory of Molecular and Cellular Biology, Hebei Collaboration Innovation Center for Cell Signaling and Environmental Adaptation, Hebei Key Laboratory of Molecular and Cellular Biology, College of Life Sciences, Hebei Normal University, Shijiazhuang, China

**Keywords:** Low temperature, Arabidopsis, miRNAs, siRNAs, lncRNAs

## Abstract

Low temperature (LT) is an important factor limiting plant growth and distribution. Plants have evolved sophisticated adaptive mechanisms to cope with hypothermia. RNA silencing is the orchestrator of these cellular responses. RNA silencing, which modifies gene expression through noncoding RNAs (ncRNAs), is a strategy used by plants to combat environmental stress. ncRNAs, which have very little protein-coding capacity, work by binding reverse complementary endogenous transcripts. In plants, ncRNAs include small non-coding RNAs (sncRNAs), medium-sized non-coding RNAs (mncRNAs), and long non-coding RNAs (lncRNAs). Apart from describing the biogenesis of different ncRNAs (miRNAs, siRNAs, and lncRNAs), we thoroughly discuss the functions of these ncRNAs during cold acclimation. Two major classes of sncRNAs, microRNAs and siRNAs, play essential regulatory roles in cold response processes through the posttranscriptional gene silencing (PTGS) pathway or transcriptional gene silencing (TGS) pathway. Microarray or transcriptome sequencing analysis can reveal a large number of cold-responsive miRNAs in plants. In this review, the cold-response patterns of miRNAs verified by Northern blotting or quantitative PCR in *Arabidopsis thaliana*, rice, and many other important crops are discussed. The detailed molecular mechanisms of several miRNAs in *Arabidopsis* (miR397, miR408, miR402, and miR394) and rice (Osa-miR156, Osa-miR319, and Osa-miR528) that regulate plant cold resistance are elucidated. In addition, the regulatory mechanism of the lncRNA SVALKA in the cold signaling pathway is explained in detail. Finally, we present the challenges for understanding the roles of small ncRNAs in cold signal transduction.

## 1 miRNAs and LT stress

LT stress greatly limits plant growth and photosynthetic production. In response to this abiotic stress, plants have evolved a variety of complex adaptive strategies that function through cold acclimation pathways to reprogram gene expression, resulting in a series of physiological and metabolic changes that help plants adapt to freezing (<0°C) or chilling stress (0°C-15°C).^1,2^ Understanding the key components in the plant cold stress response can aid in the improvement of plant cold tolerance through traditional breeding strategies or gene editing. Studies have shown that the C-repeat/dehydration-responsive element binding factors (CBFs/DREB1s)-dependent pathway is the main signaling pathway through which plants respond to cold stress.^3,4^ CBFs are a class of conserved transcription factors in the APETALA2/ETHYLENE-RESPONSIVE FACTOR (AP2/ERF) superfamily. *CBF1/DREB1B, CBF2/DREB1C*, and *CBF3/DREB1A* are arranged tandemly in a single gene cluster in *Arabidopsis*.^3–7^ In the early stage of the cold response, the transcription of CBF genes is rapidly upregulated by INDUCER OF CBF EXPRESSION 1 (ICE1),^8,9^ and CBF can directly activate hundreds of downstream *COLD-REGULATED* (*COR*) genes and then improve freezing resistance by regulating the physiological and biochemical characteristics of plant cells.^10–13^

After CBF performs its function in the early stage of cold stress, it gradually decays in the middle stage of cold stress. Studies have found that CBF can bind to 14-3-3 protein and be degraded by the proteasome.^14^ In addition, the transcription levels of CBF are decreased via regulation by noncoding RNAs (ncRNAs),^15^ suggesting the importance of ncRNAs in plant cold signal regulation. ncRNAs can be divided into sncRNAs, mncRNAs and lncRNAs.^16,17^ sncRNAs have lengths ranging from 18 nt to 30 nt. According to their synthesis pathways and functions, sncRNAs can be divided into two categories: miRNAs and small interfering RNAs (siRNAs). mncRNAs (31–200 nt) include partial rRNAs (5s and 5.8s), transfer RNAs (tRNA), small nucleolar RNAs (snoRNAs), small nuclear RNAs (snRNAs), and the newly discovered small Cajal body-specific RNAs (scaRNAs) .^16,18,19^ lncRNAs (>200 nt), owing to their relatively large size, may serve as precursors for siRNA and miRNA synthesis or as scaffolds for recruitment of other biomacromolecules. sncRNA and lncRNA are more sensitive to ambient temperature variation compare with mncRNAs. So, this review mainly discusses the research progress on plant sncRNAs and lncRNAs in recent years, focusing on the biological sources, modes of action, and functions of these ncRNAs in the plant cold response.

### 1.1 Biogenesis and modes of action of miRNAs

miRNAs are usually 20–24 nt in length. By regulating the expression of target genes at the transcriptional or posttranscriptional level, miRNAs play important roles in many biological processes.^17,20–22^ To date, 428 mature miRNAs have been identified in *Arabidopsis*, and 738 mature miRNAs have been identified in rice.^23^

miRNAs are encoded by the MIR gene, which is located mainly in the intergenic region and is transcribed under the action of RNA polymerase II (RNAPII) to produce single-stranded primary miRNA transcripts (pri-miRNAs). With the help of the core components HYPONASTIC LEAVES 1 (HYL1)^24–26^and SERRATE (SE),^27^ pri-miRNA is continuously cut two times and processed into a miRNA/miRNA* double strand by the RNase III family enzyme DICER-LIKE (DCL), usually under the action of DCL1.^28,29^ Canonical miRNA processing by DCL1 in *Arabidopsis* was discovered through capture of cryogenic electron microscopy (cryo-EM) structures of DCL1-pri-mi166f.^30^

The 3ʹ end modification of the first cleavage product, precursor miRNA (pre-miRNA) also plays a very important role in the subsequent precise processing of miRNA.^31^ Song et al. performed 3ʹ RACE sequencing on a pre-miRNA in *Arabidopsis* and found extensive cytidine and uridine modification at the 3ʹ end of the pre-miRNA. The heterogeneity of the 3ʹ end of this pre-miRNA increased the accuracy of HYL1, SE and DCL1 binding.^31^ The nucleotide transferase HEN1 SUPPRESSOR1 (HESO1) is responsible for the uridylation of most pre-miRNAs and for the cytidylation of some pre-miRNAs.^31^ The other two nucleotidyl transferases, NUCLEOTIDYL TRANSFERASE PROTEIN 6 (NTP6) and NUCLEOTIDYL TRANSFERASE PROTEIN 7 (NTP7), are also responsible for the cytidylation of some pre-miRNAs. Furthermore, the uridine modification mediated by HESO1 can lead some imprecisely processed pre-miRNAs to be degraded and cannot be loaded into AGO protein.^31^

**Figure 1.**
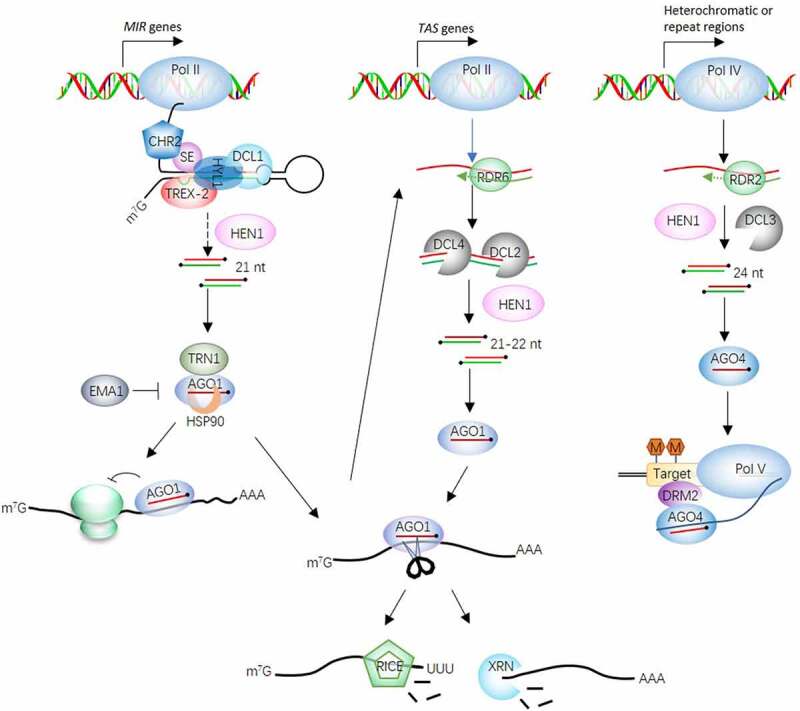
RNA silencing pathways in plants.

Pri-miRNAs and their processing-related proteins aggregate in the nucleus to form 0.2–0.8 μm dicing bodies. Recently, Yijun Qi’s research group discovered that the core component of the SE protein aggregates through weak intermolecular interactions generated by its N-terminal intrinsically disordered regions (IDRs), which is essential for driving the assembly of cleavage bodies.^32^ In addition, Xiuren Zhang’s research group found that the chromosome remodeling factor SWI2/SNF2 ATPase CHR2 can compete with SE to bind to pri-miRNA, change the conformation of pri-miRNA, and inhibit pri-miRNA processing.^33^ Therefore, pri-miRNA processing is subject to bidirectional fine adjustment. The 3ʹ-end nucleic acids of miRNA/miRNA* double strands are 2ʹ-O-methylated by HUA ENHANCER 1 (HEN1)^34,35^ to prevent the ends from being degraded. Most mature miRNA/miRNA* double strands have uracil at the 5′ end and are preferentially loaded into AGO1 of the AGO family. The miRNA* strand is removed, and finally, the miRNA-AGO1 complex is exported to the cytoplasm. In the cytoplasm, miRNAs bind to target mRNAs and guide post-transcriptional gene silencing (PTGS) under the action of miRNA-induced silencing complex (miRISC) .^36,37^ The process of miRNA loading into AGO1 is also regulated by many key factors, such as the positive regulator HSP90,^38^ TRANSPORTIN1 (TRN1),^39^ and the negative regulator ENHANCED MiRNA ACTIVITY1 (EMA1) .^40^ Recently, Xuemei Chen’s research group discovered that transcription and export complex 2 (TREX-2) plays a dual role in the positive regulation of miRNA transcription and miRNA-AGO1 complex output through the nuclear pore,^41^ indicating that the assembly process of miRISC is complex and involves multiple proteins (Figure 1).

Pri-miRNAs are transcribed by RNAPII from the relevant MIR genes and are processed by DCL1, HYL1, and SE, yielding miRNA/miRNA* duplexes. The miRNA/miRNA* duplexes are methylated by the methyltransferase HEN1. The miRNA guide strand is loaded in AGO1 and forms RISCs, which bind to the cognate targets and either arrest their translation or directly silence them. This step involves the function of HSP90. One source of pha-siRNA is RISC-cleaved RNA fragments, and another is TAS genes, the double-stranded precursors under the action of RDR6. Under the actions of DCL4 and DCL2, the double-stranded RNA is cleaved into secondary siRNAs that participate in the PTGS pathway, similar to miRNAs. Heterochromatic or repeat-associated sequences are transcribed by POL IV, and the second strand is synthesized by RDR2. The double-stranded siRNA precursors are diced by DCL3 to generate 24 nt long hc-siRNAs. hc-siRNAs participate in the TGS pathway via the actions of POL V, AGO4, and DRM2.

Plant miRNAs inhibit target gene expression through two main modes of action: transcript cleavage and translation inhibition. In the process of transcript cleavage, a miRNA recognizes a target mRNA through sequence complementation, and AGO1 directly cuts the target mRNA at the phosphodiester bond corresponding to the 10^th^ and 11^th^ nucleotides of the miRNA. This process occurs in the plant cell cytoplasm. AGO1-mediated cleavage of mRNA produces two fragments: the 5ʹ fragment and the 3ʹ fragment. The degradation of the 3ʹ fragment requires EXORIBONUCLEASE4 (XRN4), which has exonuclease activity.^42^ The 3ʹ end of the 5ʹ fragment is labeled with uridine by HESQ1 and then rapidly degraded under the action of RISC-interacting clearing exoribonucleases (RICE), which have rosette structures, to release the miRISC complex for a new cycle.^43^ In the process of miRNA-mediated translational inhibition, miRISC-mediated targeting of the 5ʹ untranslated region (UTR) can prevent ribosome recruitment and translation initiation. In contrast, miRISC-mediated targeting of open reading frames (ORFs) can prevent ribosome movement and translation extension.^44^ It is currently known that ALTERED MERISTEM PROGRAM 1 (AMP1)^45^ is a positive regulator of translation repression and colocalizes with AGO1 in the endoplasmic reticulum. The target mRNAs of miRNAs accumulate in *amp1* mutants, an effect that is particularly significant in *amp1rdr6* double mutants. However, the specific function of AMP1 has yet to be studied.

The rice genome contains 19 AGO genes. The expression of *OsAGO2* is enhanced by cold stress (fold change>2).^46^ Studies have found that LT is beneficial to the precise processing of miRNAs in *Arabidopsis*. The levels of mature miRNAs such as miRNA156 in *hyl1* and *se* mutants are significantly higher at 16 degrees than at 22 degrees, indicating that the function of DCL1 is relatively independent of HYL1 and SE.^47^ RNA sequencing of samples at 16 degrees has shown that the expression levels of 37 genes encoding proteins with nucleic acid-binding ability are significantly higher in the *hyl1* mutant than in the wild type. These 37 genes include transcription factors, DNA repair-related proteins and transcription initiation factor proteins; the role of HYL1 is difficult to replace. Further study on the secondary structures of pri-miRNAs has revealed that “GCA” and “UGCA” structures in the pri-miRNA stem pairing region are beneficial to the precise processing of pri-miRNAs at ambient LT.^47^

### 1.2 miRNA-mediated plant responses to cold stress

Research on plant miRNA expression patterns under LT stress was first carried out in *Arabidopsis*.^48–51^ The expression patterns of miRNAs under LT stress have also been reported in poplar,^52^ *Brachypodium*,^53^ rice,^54,55^ wheat,^56^ sugarcane,^57^ tomato,^58^ soybean,^59,60^ grape,^61,62^ cotton,^63^ alfalfa,^64^ eggplant^65^and *Astragalus*.^66^

The miRNA expression patterns under LT stress that have been found by high-throughput sequencing and confirmed by RNA blotting, qRT-PCR, or RT-PCR are shown in Table 1. The response patterns of miRNAs vary among different species. For example, the cold response expression pattern of miR397 differs in different plants. miR397 is upregulated in *Arabidopsis* and downregulated in grape.^48,49,61^ There are also differences in cold-responsive miRNAs within the same plant. For example, in a study on cold-responsive miRNAs in *Arabidopsis*, Liu et al. used chip technology to detect the sncRNAs in *Arabidopsis* treated at 4°C for 24 hours and found ten cold-responsive miRNAs (fold change>1.5) .^49^ However, Tiwari et al. performed an experiment at the same temperature for two days and found 107 differentially expressed (DE) miRNAs (fold change > 2) only seven of which overlapped with the miRNAs revealed by Liu et al.^51^ In addition to differences in the various detection methods, differences in plant growth status, temperature, and the duration of cold treatment may also have been responsible for the different numbers of miRNAs associated with a consistent cold response in the different articles.

**Table 1.** miRNA cold response patterns in different plants

Species	treat condition	Confirmed expression pattern of conserved miRNA	validation method	ref.
Arabidopsis thaliana	0°C for 24 h,	miR393↗ mi397b↗ miR402↗ mi319c↗ miR389a.1↘	Northern blot	^48^
Arabidopsis thaliana	4°C for 2 d	miR156a↗ miR156h↗ miR159a↗ miR167a↗ miR167c↗ miR167d↗ miR168↗ miR171a↗ miR171b↗ miR319c↗ miR393a↗ miR396a↗ miR397↗	RT-PCR	^49^
Arabidopsis thaliana	4°C for 1 h, 2 h, 6 h, 12 h, 24 h, 48 h	miR165/166↗ miR169↗ miR172a-g↗ miR396↗ miR159↗↘ miR164↗↘	Northern blot	^50^
Populus	4°C for 4 h, 8 h, 12 h, 16 h, 20 h, 24 h	miR156g-j↘ miR168a,b↘ miR475a,b↘ miR476a↘ miR477a,b↗	qRT-PCR	^52^
Brachypodium distachyon	4°C for 24 h	miR172↗ miR397↗ miR911T↘ miR926T↘ miR927T↘ miR912T↘ miR913T↘ miR914T↘ miR915T↘ miR917T↘ miR922T↘ miR928T↘ miR918T↘ miR919T↘	Northern blot	^53^
Rice	4°C for 0.5 h, 1 h, 3 h, 6 h, 9 h, 12 h, 24 h	miR167d↘ miR167e↘ miR167f↘ miR167g↘ miR167h↘ miR167i↘ miR167j↘ miR319a↘ miR319b↘	RT-PCR	^54^
Rice	5°C for 24 h	miR396↗ miR394↗ miR810b.1↗ miR810b.2↗ miRcand052↘ miR530-3p↗ miR1866↘ miR1877↘ miR1874-3p↘ miR2275d↘	Northern blot	^55^
wheat	10°C for 5d	miR167d↘ miR167c↘↗ miR172a↘ miR393↘ miR396a↘ miR444c.1↘	qRT-PCR	^56^
soybean	4°C for 24 h,	miR397a↗ miR166u↗ miR167c↗ miR171p↗ miR399i↗ miR2111f↗ miR169c↘ miR319a/b↘ miR5559↘ miR5037a↘ miR1523a↘	qRT-PCR	^59^
grapevine	4°C for 0, 2, 4, 8, 24 h	miR156↘ miR171↘ miR172↘ miR395↘miR397↘ miR398↘	qRT-PCR	^61^
cotton	4°C for 8 h	miR398b↗ miR397a2↗ miR408-5p-1↗ miR408-3p↗ miR408-5p-2↗ miR8175↗ mir_18↗ mir_3↗	qRT-PCR	^63^
Medicago sativa	4°C or −8°C for 3 h	miR160e↗ miR166f↗ miR167a↘miR172c-3p↘ miR396a-5p↘ miR5231↘	qRT-PCR	^64^
soybean	4°C for 24 h,	miR164a↗ miR4411↗ miR169e↗ miR156↘ miR167f↘	qRT-PCR	^60^
eggplant	1°C for 2, 6, 12 and 24 h.	miR168a↗ miR2652a↗ miR812v↗ miR4414a-5p↗ miR5813↘ miR167c-3p↘ miR9478-3p↘ miR4221↘ miR8577↘	qRT-PCR	^65^
grape	4°C for 4 h	miR171c↗ miR166g↘	qRT-PCR	^62^
Astragalus Membranaceus	4–5°C for 3 h, 6 h, 24 h, and 72 h.	miR168-1↗ miR169-1↗ miR397-1↗ miR2111-1↗ miR156-3↘ miR159-1↘ miR159-5↘ miR160-2↘ miR166-1↘ miR166-2↘ miR167-1↘ miR171-1↘ miR171-4↘ miR390-1↘ miR394-1↘miR396-1↘ miR396-2↘ miR398-1↘miR408-1↘ miR858-1↘ miR4415-1↘	qRT-PCR	^66^
Arabidopsis thaliana	4°C for 3 h, 6 h, and 2 d	mi163a-3p↗ miR3434-5p↗	qRT-PCR	^51^

In addition, the expression patterns of some miRNAs have different response patterns after cold stress is encountered at different developmental stages. For example, in wheat, tae-miR167c is significantly inhibited after cold stress at the L1.5 stage (at which the anther length is 1.5 mm) and upregulated after cold stress at the L3.0 stage (at which the anther length is 3 mm) .^56^ In *Arabidopsis*, miR159 and miR164 are rapidly upregulated within 1 hour of cold stress and then decrease to the basal level.^50^ Different miRNA members of the same miRNA family, due to the different cis-elements contained in their promoters, also show different expression patterns. For example, in Dongxiang common wild rice, after 6 hours of cold treatment, the expression of miR395d\k\w is increased by more than three times, while the expression of miR395e is decreased by more than twelve times.^67^ Given the spatial and temporal variability of miRNAs in the cold response, much more work is needed before full use can be made of natural or artificial miRNAs to enhance crop traits.

miR397 was one of the first miRNAs reported to be induced by cold stress.^48^ However, the specific mechanism for regulation of plant cold tolerance has not been fully resolved. *Arabidopsis* overexpressing miR397a have higher cold resistance and acquire freezing resistance.^68^ Northern blot assays have revealed that the transcript levels of CBF1 and CBF3 in miR397a-overexpressing plants are not significantly different from those in the wild type under cold treatment for 3 hours, but the transcript levels of CBF2 are significantly higher in overexpressing plants than in wild-type plants. After 48 hours of cold treatment, the transcript levels of *COR15A*, *COR47A*, *RD29A*, and other *COR* genes are significantly higher than those of the wild type.^68^ The target genes of miR397 are known to encode laccase family multicopper oxidases (LAC2, LAC4, and LAC17), which are located on the cell wall.^48,69^ Laccase can reduce the accumulation of lignin in plant cell walls and increase cell wall elasticity and permeability. Therefore, overexpressing miR397a may allows plants to endure lower-temperature stress by modulation lignification of plant cell walls.^68^ However, researchers do not understand how miR397 regulates the CBF-dependent cold signaling pathway.

In *Arabidopsis*, miR408 is a miRNA induced by various abiotic stresses, such as cold stress, oxidative stress, and salt stress. The main target gene of miR408 encodes the blue copper protein. In transgenic *Arabidopsis* overexpressing miR408, nonessential copper protein levels are reduced, which leads to increases in the transcript levels of the endogenous copper protein copper/zinc superoxide dismutases CSD1 and CSD2, which enhance the antioxidant capacity. The transcription of the copper chaperone protein CCS1 (At1g12520) also increases, enhancing the utilization of copper. Another type of target gene of miR408 includes the laccase-encoding genes *LAC3*, *LAC12*, and *LAC13*. Therefore, miR408 can also improve the cold tolerance of plants by targeting *LAC* genes.^70^ Analyses of transgenic plants overexpressing miR408 have shown that overexpression of miR408 can improve plant tolerance to salt, LT, and oxidative stress.^71^

In *Arabidopsis*, miR402 is a cold-induced miRNA.^51^ Overexpression of miR402 accelerates seed germination and promotes seedling growth in *Arabidopsis* under LT stress. *DEMETER-LIKE PROTEIN3* (*DML3*) may be one of the target genes of miR402. DML3 is a 5-methylcytosine DNA glycosidase that is involved in the control of DNA methylation status. In miR402-overexpressing transgenic plants, the expression of *DML3* is decreased, which further indicates that *DML3* is the target gene of miR402. It is speculated that miR402 may target *DML3* to regulate the adaptation of plants to cold stress. Therefore, epigenetic changes in DNA methylation status may trigger downstream signaling cascades that affect the cold tolerance of plants.^72^

Song et al. found that miR394 not only responds to drought stress and salt stress but also responds to LT stress.^73^ The abundances of pre-miR394a and pre-miR394b increase by 1.3 times and 1.8 times, respectively, after 6 hours of cold treatment at 4°C. miR394 is a highly conserved miRNA in plants, and miR394 and its target gene *LEAF CURLING RESPONSIVENESS* (*LCR*) are involved in leaf morphological development.^74^ Interestingly, *LCR* is also induced by LT. qRT-PCR analysis of *pLCR::GUS* transgenic plants has shown that LCR transcript levels are somewhat lower than those of GUS. It is speculated that some *LCR* transcripts are degraded through the posttranscriptional gene silencing (PTGS) pathway mediated by miR394.^75^ In addition, overexpression of MIR394a or MIR394b improves the freezing resistance of transgenic *Arabidopsis*. The T-DNA insertion mutants *lcr-1* and *lcr-2* also improve freezing resistance. In contrast, overexpression of *m5LCR* (m5LCR is a mutant LCR that cannot be cut by miR394) reduces the freezing resistance of *Arabidopsis*, indicating that miR394 is a positive factor that transduces cold signals in *Arabidopsis* through the target gene *LCR*.^75^ After cold treatment for 3 h and 6 h, the expression levels of *CBFs* are higher in miR394-overexpressing plants or *lcr* mutants and lower in m5LCR-overexpressing transgenic *Arabidopsis* than in wild-type plants, indicating that miR394-LCR may be involved in CBF-dependent cold acclimation pathways to regulate the freezing resistance of plants.^75^ However, the mechanism needs to be further studied.

miR165/166 is also induced by LT in *Arabidopsis*. Its target genes encode homeodomain leucine zipper class-III transcription factor family members, including *PHBULOSA* (*PHB*) and *ATHB-8*.^50^ Among them, PHB plays an important role in the differentiation of apical meristems.^76^ *ATHB-8* can be induced by auxin to promote the formation of xylem and accelerate the differentiation of vascular bundles.^50^ Jian-Kang Zhu’s research team found that using a short tandem target mimicry (STTM) to reduce the transcription of miR165/166 can increase drought and LT tolerance in transgenic plants.^77^ Further research has found that PHB can directly regulate the expression of the *ABI4* gene, and PHB can also directly activate the expression of the *BETA-GLUCOSIDASE 1* (*BG1*) gene by binding its promoter. Therefore, in STTM165/166 plants, the transcript levels of the miR165/166 target gene PHB are increased, and the expression levels of the downstream gene *BG1* are also increased. Consequently, abscisic acid-glucose ester (ABA-GE) is hydrolyzed to produce ABA, which improves drought resistance.^77^ STTM165/166 plants also have good frost resistance. Although CBFs are not altered in STTM165/166 transgenic plants, the transcript levels of the CBF-dependent *COR* genes *RD29A* and *COR15A* are increased in transgenic plants.^77^

In *Arabis alpina*, a perennial relative of *Arabidopsis*, miR156 targets the *SQUAMOSA PROMOTER BINDING PROTEIN-LIKE* (*SPL*) gene to regulate flowering under LT conditions.^78^ Recently, researchers have found that overexpression of OsmiR156 can improve cold tolerance in transgenic *Arabidopsis*, rice, and pine.^79^ The transcript levels of *OsWARKY71* are decreased in transgenic rice overexpressing OsmiR156. It is predicted that the target gene of OsmiR156 is a type of *SPL* gene. A luciferase reporter assay has shown that OsSPL3 can interact with the promoter of *OsWARKY71* to promote its transcription. Therefore, in OsmiR156-overexpressing transgenic rice, the transcript levels of *OsSPL3* and *OsWARKY71* are significantly reduced, which indirectly leads to declines in *OsWARKY71* transcript levels. In plants, the transcription factor WARKY negatively regulates the expression of MYB transcription factors. Overexpression of *OsWARKY71* downregulates two important transcription factors (OsMYB2 and OsMYB3R-2), and the main downstream genes of OsMYB2 are *OsLEA3, OsRab16A*, and *OsDREB2*. Therefore, in *OsWARKY71*-overexpressing transgenic plants, the transcript levels of *OsLEA3* and other genes are decreased significantly, and the cold tolerance of the plants is also decreased. In OsmiR156-overexpressing transgenic rice, the transcript levels of genes such as *OsLEA3* are increased, and the cold tolerance of the plants is also increased.^79^

Overexpression of Osa-miR319b can improve cold tolerance in rice.^80^ There are five predicted target genes of Osa-miR319b. Two of the target genes, *PROLIFERATING CELL FACTOR 6* (*OsPCF6*) and *TEOSINTE BRANCHED1/CYCLOIDEA/PCF 21* (*OsTCP21*), encode proteins that belong to the TCP family of transcription factors. OsPCF6 and OsTCP21 are located in the nucleus. In *OsPCF6-* and *OsTCP21*-overexpressing transgenic plants, the proline content is reduced, reactive oxygen species levels are increased, and the transcription of OsDREB1A is decreased. Therefore, OsPCF6 and OsTCP21 are negative regulators of cold acclimation in rice. In plants overexpressing Osa-miR319b, inhibition of *OsPCF6* and *OsTCP21* leads to increases in *OsDREB1A* transcript levels, thereby improving the cold tolerance of the plants.^80^

Osa-miR528 overexpression can improve cold tolerance in *Arabidopsis*, pine, and rice.^81^ The target gene Os06g06050 of Osa-miR528 can activate the expression of *OsMYB30*, so Osa-miR528 can indirectly inhibit the expression of *OsMYB30*. OsMYB30 is a negative regulator of cold tolerance because OsMYB30 can bind to the promoter of the β-amylase gene to inhibit the expression of BMY family genes. Overexpression of Osa-miR528 leads to decreased expression of *OsMYB30* and increased expression of BMY family genes. BMY family genes control the starch metabolism and lead to elevation of maltose, sucrose, and fructose content. Therefore, the increased expression of BMY genes leads accumulation of maltose, and improves plant cold tolerance.^81^

**Figure 2.**
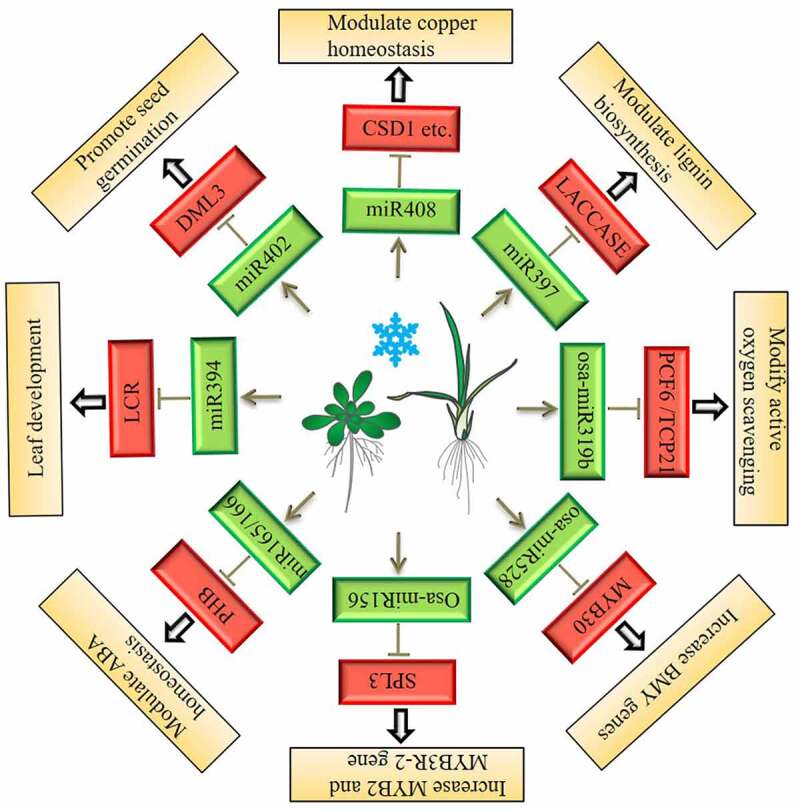
miRNA-target network module involved in cold signal transduction.

In summary, based on the miRNA response patterns of various plants, overexpression of cold-responsive miRNAs usually inhibits the negative regulatory genes of cold tolerance, thereby improving plant cold stress tolerance (Figure 2). However, some miRNAs have more than one target gene, such as miR166/165, which implies the complexity of the roles of miRNAs in regulating plant cold signal transduction.^50,77^ Thus far, most studies have focused on the stress response patterns of miRNAs in different species of plants, various mutants of the same species, or different tissues. There are only a few valuable clues that can clearly explain how miRNAs respond to and affect the transcript accumulation and translation rates of target proteins, thereby affecting the cold tolerance of plants. Among miRNAs, only a few may impact the cold tolerance of plants through the known CBF-COR pathway. Gradually, research on the CBF-COR-independent pathway will establish a theoretical basis for understanding the functions of miRNAs in plant cold adaptation.

This pathway is based on the expression trends of miRNAs and their target genes after cold stress. The green box represents upregulation, and the red box represents downregulation.

## 2 siRNAs and LT stress

### 2.1 Biogenesis and mode of action of siRNAs

siRNAs and miRNAs have similar structures and functions but different precursors. siRNAs are derived from long double-stranded RNA molecules. The common feature of siRNAs and miRNAs is that the 3ʹ end is modified by HEN1.^82^ The production of siRNAs depends on RNA-DEPENDENT RNA POLYMERASE (RDR), which uses single-stranded RNA as a template to synthesize double-stranded RNA. Double-stranded RNA is cleaved by DCLs to generate 21–24 nt siRNAs; the lengths of the siRNAs depend on the catalytic activity of the corresponding DCL, but the synthesis of miRNAs does not require RDR.^83^ According to the different sources and processing enzymes, plant siRNAs can be sorted into three main types: heterochromatic siRNAs (hc-siRNAs), phased secondary siRNAs (pha-siRNAs), and natural antisense transcript siRNAs (nat-siRNAs) .^84^

Hc-siRNAs are predominantly 24 nt in length and are also known as repeat-associated siRNAs (ra-siRNAs). Most plant endogenous siRNAs belong to this category. Single-stranded primary transcripts are transcribed by plant-specific RNA polymerase IV (Pol IV) and are derived from repeat regions or transposon regions. Double-stranded precursors are synthesized by RNA-DEPENDENT RNA POLYMERASE 2 (RDR2) and then processed by DCL3. hc-siRNAs are recognized by AGO4 to form AGO4-siRNA complexes. Since hc-siRNAs can be complementary to the ncRNA transcribed by Pol V, AGO4 is further recruited by the C terminal domain (CTD) of Pol V,^85^ and AGO4 then recruits DRM2^86^ to the transcription site of Pol V, directing the de novo methylation of DNA at that site. This biological process is called RNA-directed DNA methylation (RdDM) and eventually leads to transcriptional gene silencing (TGS) to maintain the stability and integrity of the heterochromatin genome.^83,87 46^

Pha-siRNAs are another type of endogenous siRNA. There are two sources of pha-siRNAs. One source is RISC-cleaved RNA, the 3ʹ end of which is protected by SUPPRESSOR OF GENE SILENCING 3 (SGS3). The second strand is synthesized under the action of RNA-DEPENDENT RNA POLYMERASE 6 (RDR6) and then cut into 21 nt-22 nt siRNAs under the action of DCL4 or DCL2 protein. Another source involves transcription and processing from the TAS gene. The resulting siRNAs are called trans-acting siRNAs (tasiRNAs), which are 21-nt pha-siRNAs that rely on DCL4 and are produced from noncoding TAS transcription products. Four families of trans-acting siRNA (TAS) genes have been identified, TAS1 to TAS4. TAS1 and TAS2 are recognized by miR173, and TAS3 and TAS4 are recognized by miR390 and miR173, respectively.^84,88^ These miRNAs guide the AGO protein to cut the primary transcript and then generate double-stranded precursors under the action of RDR6; the precursors are cleaved into mature tasiRNAs under the action of DCL4. In addition to TAS, in dicotyledonous plants, pha-siRNAs can also be produced from protein-coding genes, such as *NUCLEOTIDE-BINDING LEUCINE-RICH REPEAT* (*NB-LRR*) and *PENTATRICOPEPTIDE REPEAT* (*PPR*) genes. Unlike hc-siRNAs, pha-siRNAs regulate the expression of target genes at the posttranscriptional level.

Nat-siRNAs are produced in the natural antisense transcription pairing region. Generally, one of the genes is constitutively expressed and the other is inducible,^89^ but the synthesis process of nat-siRNAs is not well understood.^90^ In *Arabidopsis*, it is found that nat-siRNAs can be synthesized from *SIMILAR TO RCD ONE 5* (*SRO5*) and *DELTA-1-PYRROLINE-5-CARBOXYLATE DEHYDROGENASE* (*P5CDH*) natural cis-antisense double-stranded RNA.^91^ The 21-nt P5CDH nat-siRNAs reduce the transcription of the *P5CDH* gene through mRNA cleavage via the PTGS pathway.

### 2.2 siRNA-mediated plant responses to LT stress

In addition to playing a role in plant growth and maintaining genome integrity, siRNAs are also important components in the plant stress response. In 2018, the Marquardt research group of the University of Copenhagen found that the expression level of the *CBF1* gene in an *rdr6* mutant was much lower than that of the wild type under cold stress and almost undetectable in a *dcl3* mutant, suggesting that the siRNA pathway may be involved in the regulation of CBF-dependent pathway.^15^ Recently, researchers found that pha-siRNA derived from the *PPR* gene (AT1G63070) was upregulated after 6 hours of cold treatment in *Arabidopsis*, and one of the predicted target genes, *PPR gene* (AT1G18485), was downregulated after LT stress.^51^ Yao et al. found that in wheat seedlings, cold, heat, salt, or drought stress significantly changed the expression of four nat-siRNAs. Among them, nat-siRNA 005047_0654_1904.1 was significantly upregulated under LT stress and downregulated under other abiotic stresses.^92^ However, the function of nat-siRNA 005047_0654_1904.1 in LT stress signaling remains to be studied. Recently, 104 cis-nat-siRNAs and 38 trans-nat-siRNAs in *Arabidopsis* were found to be upregulated or downregulated at different time points during LT stress (from 3 hours to 2 days) .^51^ qRT-PCR verified that the production of cis-nat-siRNA from the AT3G05870-AT3G05880 transcript and trans-nat-siRNA derived from the AT1G10522-AT5G53905 transcript could be induced by LT stress, and the peak value occurred after approximately 6 hours of exposure to LT stress.^51^ The functions of these two nat-siRNAs in LT stress signal transduction need to be studied.

Compared with that of the roles of miRNAs, understanding of the roles of siRNAs in plant LT stress is still in its infancy. Moreover, the sources of siRNAs are complex. Many secondary siRNAs are based on the production and action of miRNAs. All of these factors make the cold response patterns of siRNAs in different laboratories less reproducible than the cold response patterns of miRNAs. However, the RdDM pathway, in which most siRNAs participate to control gene silencing at the genome level, is more direct and economical. Moreover, offspring can inherit the epigenetic modifications of genes, which has played a vital role in cold domestication during plant evolution.

## 3 lncRNAs and LT stress

### 3.1 Biogenesis and modes of action of lncRNAs

lncRNAs are a type of RNA longer than 200 nucleotides and have no obvious protein-coding ability. Similar to mRNAs, most lncRNAs are transcribed from the 5ʹ end by RNAPII and then 5ʹ-capped, spliced and 3ʹ-polyadenylated. A small portion of lncRNAs and nonpolyadenylated lncRNAs are transcribed by RNA polymerase III. lncRNAs share many common features with mRNAs, such as posttranscriptional processing, promoter characteristics and RNA structure formation. Many lncRNAs show spatiotemporal specificity, tissue specificity, and cell-specific expression. Compared with mRNAs, lncRNA transcripts are shorter and lack many motifs, such as ORFs and Kozak consensus sequences.

Compared with mRNAs, lncRNAs have lower expression, but the expression variability is higher. lncRNAs lack sequence conservation between species and show a low degree of evolutionary conservation across species.^93^ When an lncRNA-coding sequence is transcribed by RNAPII, the transcription unit can affect the transcription efficiency of neighboring genes.^15^ In addition, mature lncRNAs have two molecular functions: 1) they can be used as precursors to synthesize miRNAs or siRNAs, and 2) as scaffolds, they can bind to DNA, RNA and proteins (or protein complexes) to execute diverse functions at the epigenetic, transcriptional, or posttranscriptional levels.

### 3.2 lncRNA-mediated plant responses to LT stress

The cold response of lncRNAs in *Arabidopsis*,^51,94^ rice,^95^ alfalfa,^96^ and banana^97^ has been studied and sequentially described. The cold response of lncRNAs is different from that of miRNAs and siRNAs. In addition to cold-induced differential gene expression (DE), cold-induced differential alternative splicing (DAS) has also been observed. The latest research results from Calixto et al. in *Arabidopsis* showed that nearly one-third of the lncRNAs had cold responses, including 113 DE lncRNAs and 46 DAS lncRNAs, and the two types of cold-responsive lncRNAs had an overlap of 24 DE+DAS lncRNAs.^98^ Because the expression of lncRNAs is highly dependent on tissue type and developmental stage coupled with the specific experimental system used, the reproducibility between different studies is poor. For example, among the 7,231 rice lncRNAs sequenced by Jiapei Yuan et al. in 2018, 46% were newly discovered lncRNAs. In their sequence results, there were 135 LT-responsive lncRNAs, including 29 LT-induced lncRNAs and 106 LT-repressed lncRNAs.^95^ The related molecular mechanism has not yet been elucidated.

lncRNAs play an important role in the vernalization process of plants. One of the main epigenetic changes caused by vernalization is the silencing of *FLOWERING LOCUS C* (*FLC*) genes. The silencing of FLC is mediated by the evolutionarily conserved molecule POLYCOMB REPRESSION COMPLEX 2 (PRC2) .^99^ In the early stage of vernalization, lncRNA COLDAIR recruits PRC2 in the first intron region of FLC, leading to H3K27me3 modification of histones. Subsequently, another lncRNA, COLDWRAP, recruits PRC2 in the FLC promoter region, expanding the scope of histone H3K27me3 modification.^100,101^ In vitro and in vivo experiments have shown that mutant COLDWRAP cannot bind to PRC2. Therefore, the structural integrity of lncRNAs is necessary for the normal function of lncRNA-PRC2 in the body. This cold-triggered lncRNA cascade establishes lasting and stable inhibition of the FLC gene.^102^

Recently, the Marquardt research group found that the lncRNA SVALKA can finely regulate the expression of the CBF1 gene in the mid-stage of LT stress.^15^ In *Arabidopsis*, endogenous CBF1 transcript levels peak in a short time during the cold acclimation process and then decline, which indicates the importance of strict regulation of CBF1. The Marquardt research team found a low-temperature-inducible lncRNA through transcription start site sequencing (TSS-seq) that is transcribed on the antisense strand in the intergenic region between CBF3 and CBF1 and named it SVALKA. The transcription of the lncRNA SVALKA generates antisense CBF1 lncRNA (asCBF1). HUA ENHANCER 2 (HEN2) is part of the nucleoplasm 3ʹ to 5ʹ exosome and is responsible for degrading many types of ncRNAs. Approximately 250 nt of asCBF1 can be detected in a *hen2* mutant but not in the wild type. The transcript of asCBF1 can be identified by RNA polymerase II (RNAPII) immunoprecipitation, but it cannot be detected in mature RNA. Therefore, 250 nt asCBF1 may be processed by exosomes to generate the lncRNA SVALKA during the posttranscriptional maturation process. Researchers have found that after 8 hours of LT stress at 4°C, the occupancy rate of RNAPII in the CBF1 promoter region is decreased, and the occupancy rate of RNAPII in the exon of CBF1 is higher after 8 hours of LT stress than after 4 hours of LT stress. In addition, on the complementary strand of the CBF1 3ʹ UTR, the RNAPII occupancy rate belonging to lncRNA SVALKA also increases rapidly. Therefore, it is speculated that the transcription of CBF1 and SVALKA causes the collision of RNAPII in the opposite directions, thereby limiting the transcription efficiency of full-length CBF1. This work reveals the significant roles of lncRNAs in the process of cold acclimation signal transduction.^15^

The functions of lncRNAs as precursors of sncRNAs in LT remain to be explored. In addition, some non-cold-responsive constitutively expressed lncRNAs also play roles in the stress response. However, most of the functions of lncRNAs have not been clearly explained, so related research will likely continue.

## 4 Opportunities and challenges

This review mainly introduces the functions of miRNAs, siRNAs and lncRNAs in LT stress. Although circRNAs exist in many species, they cannot be analyzed by direct sequencing of the transcriptomic poly(A)-tails due to their non-poly(A) and noncollinear structural characteristics. CircRNAs were not discovered through specific RNA sequencing methods until recent years.^103^ CircRNAs are expressed in a cell-type and tissue-specific manner in plants and are more conserved than linear lncRNAs. Furthermore, their abundance is extremely low. Although cold-induced circRNAs have recently been found in tomatoes^104^ and soybeans^105^ through large-scale sequencing, further research is needed to reveal the regulatory roles of circRNAs in plant abiotic stress.

A variety of new ncRNAs in model plants are still being discovered,^51^ indicating that the cloning of ncRNAs in plants is not yet complete and that more cold-responsive ncRNAs need to be explored. Although many miRNAs and siRNAs have been found through high-throughput sequencing to respond to plant LT stress, the specific mechanisms of action of these RNA molecules remain relatively unclear. Through forward genetic screening^41,106^ and immunoprecipitation,^43,107^ new signal elements involved in the synthesis, transport, and degradation processes of miRNA and siRNA are being discovered, which provides a research basis for elucidating the roles of small RNA molecules in plant LT stress.
